# Measuring haemolysis in cattle serum by direct UV–VIS and RGB digital image-based methods

**DOI:** 10.1038/s41598-022-17842-4

**Published:** 2022-08-08

**Authors:** Belén Larrán, Marta López-Alonso, Marta Miranda, Víctor Pereira, Lucas Rigueira, María Luisa Suárez, Carlos Herrero-Latorre

**Affiliations:** 1 Department of Anatomy, Animal Production and Clinical Veterinary Sciences, Faculty of Veterinary, University of Santiago de Compostela, Campus Terra, 27002 Lugo, Spain; 2 Veterinary Teaching Hospital “Rof-Codina”, Faculty of Veterinary, University of Santiago de Compostela, Campus Terra, 27002 Lugo, Spain; 3 Department of Animal Pathology, Faculty of Veterinary, University of Santiago de Compostela, Campus Terra, 27002 Lugo, Spain; 4 Research Institute on Chemical and Biological Analysis, Analytical Chemistry, Nutrition and Bromatology Department, Faculty of Sciences, University of Santiago de Compostela, Campus Terra, 27002 Lugo, Spain

**Keywords:** Biochemical assays, Optical spectroscopy

## Abstract

A simple, rapid procedure is required for the routine detection and quantification of haemolysis, one of the main sources of unreliable results in serum analysis. In this study, we compared two different approaches for the rapid determination of haemolysis in cattle serum. The first consisted of estimating haemolysis via a simple direct ultraviolet–visible (UV–VIS) spectrophotometric measurement of serum samples. The second involved analysis of red, green, blue (RGB) colour data extracted from digital images of serum samples and relating the haemoglobin (Hb) content by means of both univariate (R, G, B and intensity separately) and multivariate calibrations (R, G, B and intensity jointly) using partial least squares regression and artificial neural networks. The direct UV–VIS analysis and RGB-multivariate analysis using neural network methods were both appropriate for evaluating haemolysis in serum cattle samples. The procedures displayed good accuracy (mean recoveries of 100.7 and 102.1%, respectively), adequate precision (with coefficients of variation from 0.21 to 2.68%), limit of detection (0.14 and 0.21 g L^–1^, respectively), and linearity of up to 10 g L^–1^.

## Introduction

Haemolysis is the disruption of erythrocyte membranes with the subsequent release of the cell contents into the surrounding fluid. It can occur due to inadequate sample handling or to pathology. Haemolysis commonly occurs in vitro due to incorrect sample extraction, transport, preparation or storage. In vivo haemolysis is less frequent and occurs before blood collection owing to pathological conditions such as infections, toxic effects, immune-mediated diseases, inherited red blood cell disorders, disseminated intravascular coagulation and mechanical and other factors^1^. In vivo haemolysis can have serious consequences for patients, and testing is therefore carried out with the aim of distinguishing between in vivo and in vitro haemolysis, as any suspicion of pathological origin must be investigated further.

Regardless of the origin, haemolysis is the leading cause of sample rejection in human clinical pathology^2^ and it is also likely to be a frequent error in veterinary medicine^3^. Haemolysis may modify analytical results via the release of intracellular components into plasma, the dilution of the sample or producing spectrophotometric or chemical interference^4^. The results of routine blood analysis can vary depending on the degree of haemolysis, the animal species involved and the method and instrument used in the analysis. Miscalculations due to haemolysis have been reported for diverse analytes such as alanine aminotransferase (ALT), aspartate aminotransferase (AST), g-glutamyltransferase (GGT), creatine kinase (CK), lactate dehydrogenase (LDH), lipase, total bilirubin, albumin, glucose, creatinine, urea, calcium, copper, iron, magnesium, molybdenum, selenium, zinc, potassium, sodium and chloride^5–11^. The magnitude of difference is highly variable, as for some analytes the relationship with the degree of haemolysis is linear, as with iron, zinc, potassium and bilirubin, while e.g. calcium and chloride show a non-linear relationship, which could lead to significant measurement errors^1,7^. In addition, haemolysis can also interfere with immunoassays and coagulation tests^12,13^. Preanalytical control of haemolysis is therefore good practice.

Visual detection is the simplest way of determining haemolysis in a sample, as the colour varies depending on the degree of haemolysis. However, several studies^14,15^ have demonstrated that visual examination is an unreliable means of evaluating haemolysis and that this practice could affect clinical decisions^16^. Thus, although a concentration of free haemoglobin (Hb) ≥ 0.5 g L^–1^ is considered clinically significant for the most haemolysis sensitive assays^17^, some researchers consider that reliable visual detection of concentrations less than 2 g L^–1^ may be difficult^18^ because of the individual variations in the colour of serum. Hence, visual inspection of samples could produce biased results, and some analytes like LDH and AST would be affected even when a Hb concentration in serum of less than 0.5 g L^–1^ is detected^8^. For these reasons, quantification of haemolysis is important in order to prevent unnecessary sample rejection. Objective measurements must therefore be used to determine whether samples should be rejected or accepted on the basis of the required threshold of acceptability for each type of analysis. Moreover, evaluation of haemolysis could also be useful for indicating the direction of any bias in the parameters, even though the use of corrective formulas is generally discouraged^2^.

The need to estimate the degree of haemolysis has driven manufacturers of automated devices (particularly in clinical biochemistry) to develop techniques based on the haemolysis index (HI). The HI is a measurement related to the red colour of serum, which is almost exclusively caused by Hb derived from ruptured red blood cells. However, the HI is estimated in different ways in the different devices available, and there is a lack of harmonization of the methods used to detect and quantify haemolysis by different manufacturers^19^. In addition, smaller, less well equipped laboratories and laboratories where blood samples are only occasionally analyzed may not have automated devices for measuring HI. Thus, the main objective of the present study was to develop a simple method for quantifying the degree of haemolysis in serum samples in laboratories where automated devices are not available. Two different methods were assayed in cattle serum samples. The first method is based on the direct measurement of the colour of serum by ultraviolet–visible (UV–VIS) spectrophotometry, as most laboratories usually have the spectrophotometric equipment. The second methods to estimate haemolysis are based on RGB data from digital images of serum samples. The RGB information is extracted from digital pictures of the samples using a free image processing software, and from these data the prediction of the degree of hemolysis is made by means of partial least squares regression or an artificial neural network. This system of measuring haemolysis could potentially be used by small field laboratories where spectrophotometric equipment is not available, or by clinicians who need to determine the suitability and quality of serum samples before sending them to a specialized laboratory, as incorrect categorization of samples is the main cause of haemolysis in the preanalytical phase^1^.

## Material and methods

### Ethics statement

Data collection was carried out according to Directive 2010/63/EU on the protection of animals used for scientific purposes^20^, and the trial complied with the Spanish legislation on animal care^21^. The procedures were approved by the Bioethics Committee of the Rof-Codina Veterinary Teaching Hospital, University of Santiago de Compostela (Spain) (AELU001/21/INVMED(02)/Animal(05)/MM/01). The study is reported in accordance with ARRIVE guidelines.

### Blood samples

Blood was obtained from the jugular vein in ten healthy Holstein–Friesian cows used for teaching clinical examination methods and housed in the Rof-Codina Veterinary Teaching Hospital, Faculty of Veterinary Medicine, University of Santiago de Compostela (Spain).

Three types of blood samples were collected from each cow, to obtain serum, to prepare the haemolysate and for haematological analysis. Whole blood samples collected in 9 mL serum tubes (Vacuette®, CAT Serum Clot Activator; Greiner bio-one, Kremsmünster, Austria) were centrifuged at 1500*g* for 15 min within 4 h of collection to yield serum. The tubes of serum were stored at –20 ºC for further analysis. The haemolysate was obtained by freezing (–20 ºC) whole blood samples collected in 9 mL tubes containing sodium heparin (Vacuette®, NH sodium heparin, Greiner bio-one, Kremsmünster, Austria). Haematological analysis was performed in whole blood samples collected in 6 mL tubes containing ethylenediamine-tetraacetic acid (EDTA) (Vacuette^®^, K2E EDTA K2, Greiner bio-one, Kremsmünster, Austria).

**Figure 1:**
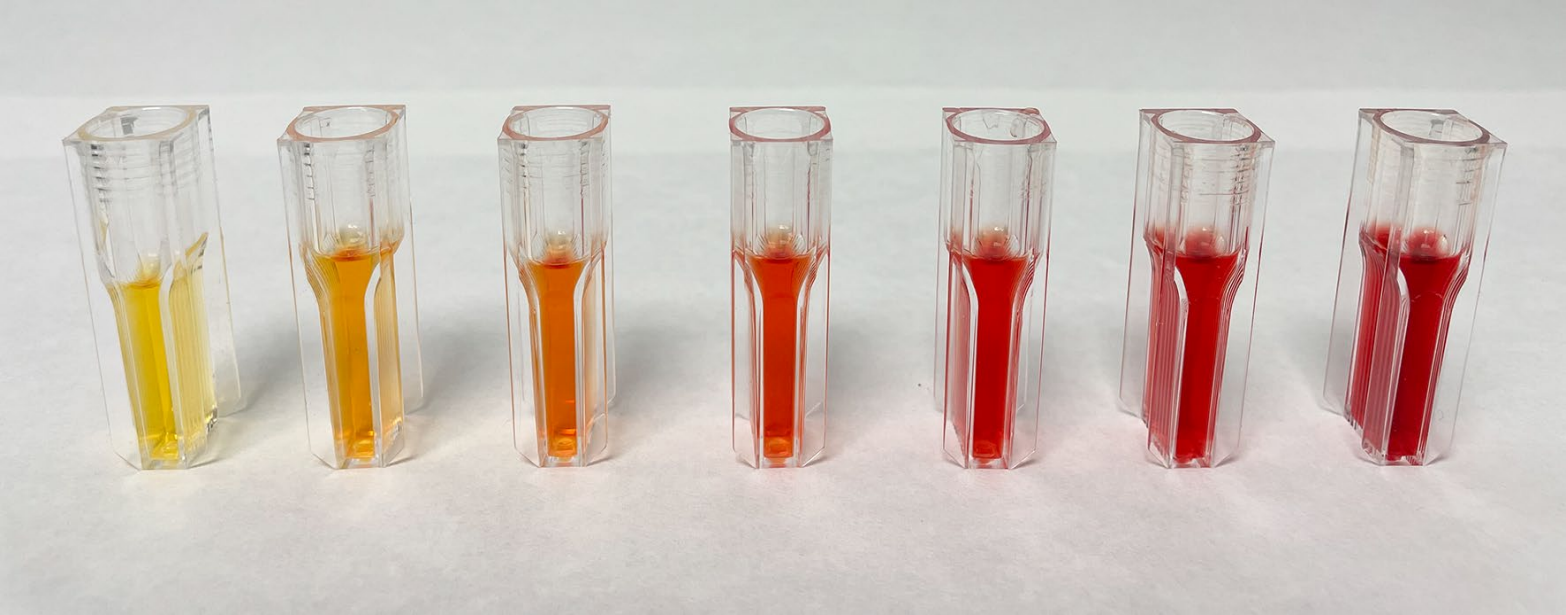
Color of serum samples containing different haemoglobin (Hb) concentrations (from the left to the right: 0.0, 0.2, 0.5, 1.0, 2.5, 5.0 and 10 g L^–1^, respectively). (Taken in our laboratory at the Faculty of Veterinary Medicine of the University of Santiago de Compostela on May 24, 2022 by C. Herrero Latorre using an Apple Iphone 12).

**Figure 2:**
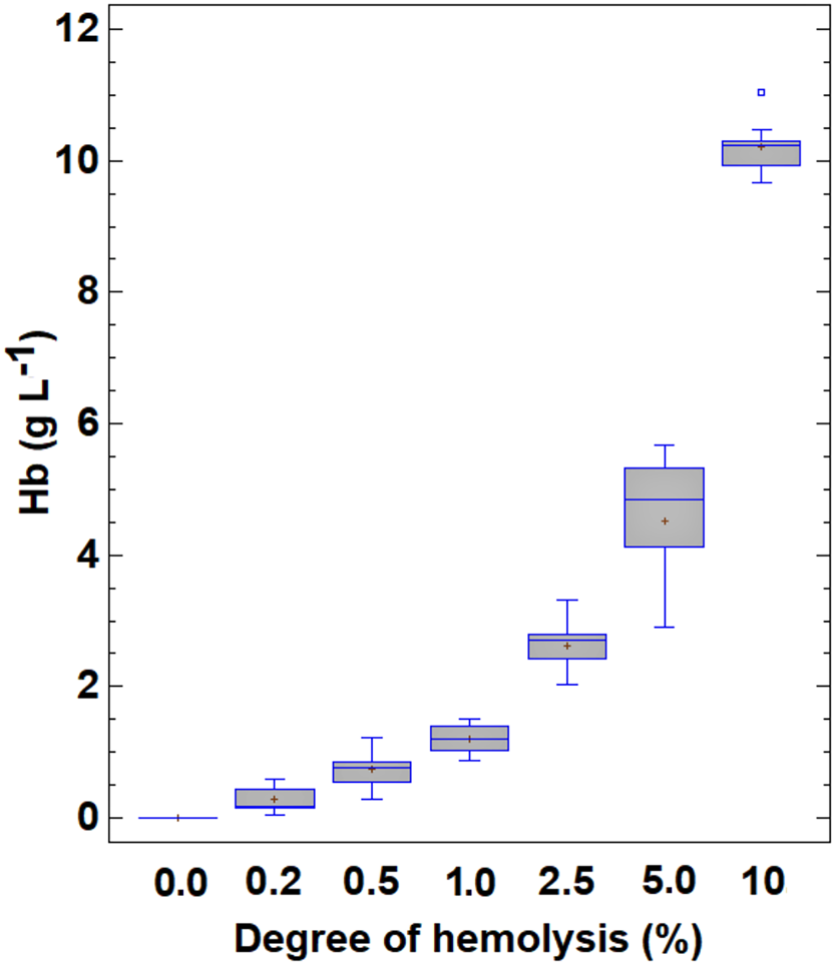
Box and whisker-plot of the serum haemoglobin (Hb) concentration in relation to the degree of haemolysis of the samples.

Each serum sample was divided into seven subsamples, to which increasing amounts of haemolysate were added to produce 0.0%, 0.2%, 0.5%, 1.0%, 2.5%, 5.0% and 10% haemolysis (Fig. 1). The degree of haemolysis, expressed as grams of Hb per litre, was calculated from the concentration of Hb of each whole blood sample determined in an automated blood cell counter. The relationship between the Hb concentration in the samples, including the individual superimposed points and the degree of haemolysis in the range 0.0–10% is shown in a box-and-whisker plot (Fig. 2). The concentration of Hb varies slightly with the degree of haemolysis owing to the natural variability in the Hb content and free blood Hb in erythrocytes from different individuals. Therefore, a total of 70 graded haemolysis samples were used in the different methods proposed.

### Apparatus and software

An automated blood cell counter (ProCyte Dx, IDEXX Laboratories, Westbrook, Maine, USA), based on a combination of fluorescence laser flow cytometry and laminar flow impedance technologies, was used to produce complete blood counts. The haematological parameters were normal in all of the cows^22^. Spectrophotometric UV–VIS measurements were carried out in a Thermo Scientific Genesys 6 spectrophotometer (Thermo Electron Corporation, Madison, USA). Blood samples were centrifuged in an iFuge-D06 centrifuge (Neuation Technologies, Gujarat, India).

Digital images were obtained in an in-house system described in detail in a previous paper^23^. Briefly, the system comprises a digital reflex camera (Canon-50D) operated under controlled light emitted from a white LED. The camera was equipped with a Sigma 105 mm f/2.8 macro lens, and it was operated remotely from a notebook computer with the control software supplied by the manufacturer (EOS Utility 2.14.10). Digital images were processed to obtain RGB data with *ImageJ* 1.52a software, developed by the National Health Institutes, USA (available free at http://imagej.nih.gov/ij). Univariate and partial least square regression multivariate calibration procedures and other statistical calculations were performed with Statgraphics Centurion XVIII, version 18.1.12 (Statistical Graphics, Rockville, Madison, USA). Neural-networks were developed using WinNN32, version 1.6a (Y. Danon. Arad, Israel). Other statistical calculations (as the joint test for slope and intercept) were carried out using the library *Ellipse* on the free software environment for statistical computing and graphics *R*, version 4.0.5 (R Foundation for Statistical Computing, Vienna, Austria).

### Analytical procedures

#### Determination of the concentration of haemoglobin by the cyanmethemoglobin method

Haemoglobin is the main intracellular molecule in red cells and is therefore the best indicator of the degree of haemolysis in blood samples. The concentration of Hb in the graded haemolysis samples was measured by the cyanmethemoglobin method (the international reference method for measuring Hb concentration) with a laboratory assay kit (Spinreact, Girona, Spain). Basically, Hb is oxidized by potassium ferricyanide into methemoglobin, which is converted by potassium cyanide into cyanmethemoglobin (CNMHb). The intensity of the colour formed (measured at 540 nm by UV–VIS spectrophotometry) is directly proportional to the Hb concentration in the sample. The results obtained using the proposed methods described in the two following sections were compared with those generated by this CNMHb-based procedure.

#### Direct UV–VIS spectrophotometric method

Haemoglobin can be estimated by direct spectroscopic measurement of serum samples at 540 nm, which is one of the major absorbance peaks of this compound and its derivatives. In the present study, this direct measurement method was applied to dilutions (1:10) of the graded haemolysis samples.

#### RGB method

This method is based on the colour information provided by single digital images of the samples. Haemolyzed serum samples were placed in spectrophotometer cuvettes (1.75 mL), which were held at a fixed distance (15 cm) from the focal plane of the camera. In all cases, images were captured using manual focus, targeting the central point of the cuvette, with the following settings: fixed custom white balance (5200 K), aperture f2.8, exposure time 1/10 s and 100 ASA photographic sensitivity. Images were obtained by remote, computer-based operation to prevent undesired movements of the camera, and all images were directly stored in the hard disc of the computer as uncompressed *jpeg* files. Previous experiments demonstrated that *jpeg* format is preferable to other formats such as *raw* or *tiff* files^23^. The RGB histograms extracted from *jpeg* files retained the colour information in smaller files than *raw* or *tiff* files, allowing faster handling and taking up much less disk space. The R, G, B and weighted intensity values were extracted from the photographs with *ImageJ*, an open-source image processing program designed for multidimensional images. For each sample, the RGB values, indicating the intensity of the red, green and blue colours, were extracted from each image with *ImageJ* software. Each intensity value is expressed on a scale of 0 to 255 (256 channels) where higher channel numbers indicate lighter colours. The colour value, considered the analytical signal, is related to the concentration of Hb. In the case at hand, two different calibration procedures were assayed: i) univariate calibration with each of the R, G, B and intensity values; and ii) multivariate calibration based on all four R, G, B and intensity variables jointly by means of partial least squares regression (PLSR) and multilayer feed-forward artificial neural network (MLF-ANN).

### Data matrix, calibration and chemometric procedures, and quality control

A data matrix (X_70x7_) was constructed from the sample data. In all cases, rows (70) corresponded to haemolyzed serum samples and the columns (7) to the numerical values of the seven variables characterizing the samples: (1) the sample code; (2) the value of Hb determined by the CNMHb method; (3) the absorbance of the samples measured at 540 nm by UV–VIS spectrophotometry; and (4–7) respectively the values of R, G, B and the weighted intensity obtained from the digital images with *ImageJ* software.

The data matrix was subjected to different univariate and multivariate chemometric calibrations to explore the relationship between the Hb concentration and the analytical signal(s) considered. In the direct UV–VIS spectrophotometric method, univariate calibration was carried out for the absorbance at 540 nm and the value of Hb determined by CNMHb. In the RGB procedures, both univariate and multivariate calibration were assayed. In the univariate approach, the R, G, B and weighted intensity values were plotted against the value of CNMHb-Hb. For multivariate calibrations, a set of four predictor *X-*variables (R, G, B and weighted intensity) and one dependent *Y-*response (Hb content) were considered. Two different approaches (PLSR and MLF-ANN) were used to construct different mathematical models for prediction. The PLSR method involves deriving the complex relationship between *X-*variables and the *Y-*response. For prediction (determined by cross-validation) a limited number of latent factors (LF) were constructed by means of PLSR. The LFs are associated with directions in the factor-space related to high variation in the *Y-*response, and they are biased to obtain the best prediction (for a more detailed explanation, see Geladi and Kowalski^24^). On the other hand, MLF-ANN is a powerful pattern recognition technique that develops models on the basis of a set of input/output samples (RGB colour data and Hb content) updating and changing the weights between neuron connections to yield an adequate output for each input. Thus, the weights of connections provide useful information about the relationship between input colour data and the output Hb content. However, this information is not chemically interpretable^25^.

Validation strategies were different for univariate and multivariate approaches. For univariate methods, the set of 70 samples was divided into calibration (49 samples: 70% of total), and validation (21 samples, 30%) subsets. Assignation of the samples to each subset was carried out at random but considering the different Hb concentrations. This sample arrangement provided enough information for the univariate models building and appropriate validation with the remaining samples. For multivariate calibrations (PLSR and MLF-ANN), the models should be constructed using as much information as possible and it is true that the optimal situation would be to have a great number of samples that would allow having enough samples to establish two independent and large sets for learning and validation. In the case at hand, due to the limited number of available samples, a nested cross-validation procedure that produces robust and unbiased performance estimates regardless of reduced sample size^26^. 90% of the samples were used to build the prediction model and the other 10% are used for validation. According to this set distribution, a tenfold nested cross-validation procedure was carried out with different constitutions of training and validation sets ensuring independence between both sets and developing a different model in each step. The overall performance was then calculated as a mean of classification performances of the 10 separately developed models on different 10% sets of the validation data which was not involved in developing the models^26^. Precision was evaluated in terms of intermediate reproducibility assays by evaluating the coefficient of variation (CV = SD/$\stackrel{\mathrm{-}}{\text{x}}$*100) for ten measures of replicates at three different concentration levels (0.5, 2.5 and 10%), prepared with different serum samples and measured different operators. The linearity of the response for all methods developed was tested for up to 10% Hb content, and the limit of detection (LOD) was calculated as 3 times and LOQ as 10 times the standard deviation of the ten (n = 10) serum blanks. Accuracy was evaluated by comparing the Hb concentrations obtained by each method developed with those obtained by the CNMHb method.

The study is in accordance with relevant guidelines and regulations.

## Results

### Direct UV–VIS spectrophotometric method

**Figure 3:**
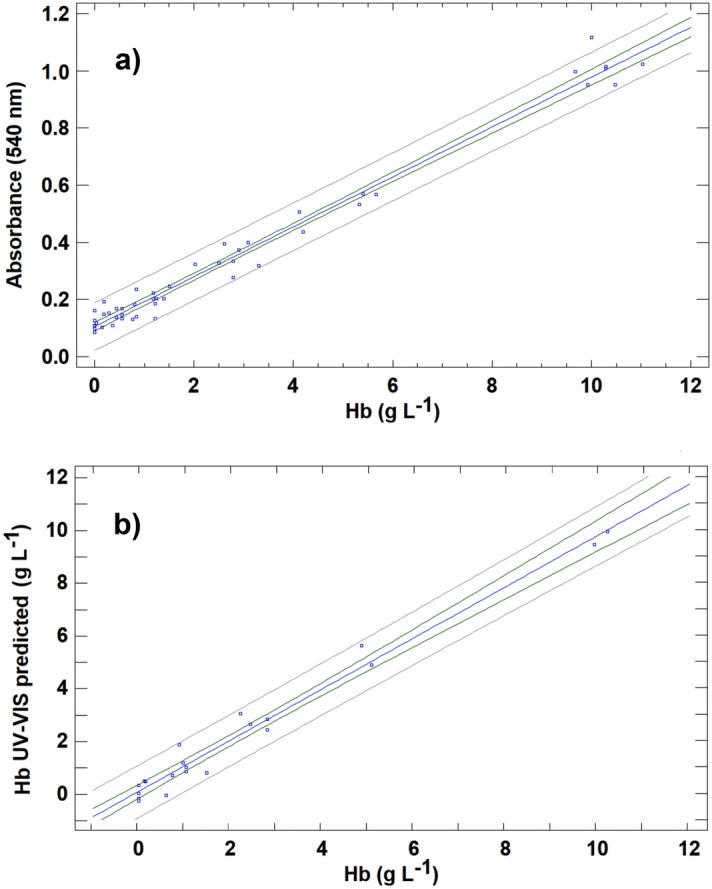
(**a**) Calibration curve for haemoglobin (Hb) concentration prepared from calibration set for the direct UV–VIS spectrophotometric method. (**b**) Prediction of haemoglobin (Hb) concentration levels using the direct UV–VIS spectrophotometric method for the validation samples compared with those provided by the CNMHb method.

The 49 calibration samples were used to confirm the relationship between the Hb concentration and absorbance at 540 nm in the cattle serum samples. The relationship follows the Lambert–Beer law, exhibiting a linear model described by Absorbance = (0.1051 ± 0.0075) + (0.0873 ± 0.0017) (Hb), with a correlation coefficient of 0.991 (Fig. 3a). This result was also checked by ANOVA, and the linear relationship between absorbance and Hb was found to be significant (*P* < 0.05).

Once the appropriate linear relationship between absorbance and the Hb concentration in cattle serum samples was demonstrated, different studies were carried out to establish the analytical figures of merit in relation to accuracy, precision, linearity, LOD and limit of quantification (LOQ) of the method. Accuracy was evaluated by predicting the Hb concentration in the 21 known samples in the validation set. After measurement of absorbance at 540 nm in these samples, the calibration curve obtained was used to predict the Hb concentration. The Hb concentration determined by UV–VIS was plotted against the concentration measured using the CNMHb method (Fig. 3b). Linear regression is probably the most common approach used to compare different analytical methods. Based on this approach, if the two assayed methods provide comparable results, the regression line between the test and the reference methods should yield a straight line that is not significantly different from the line of equality (characterized by a slope equal to 1 and an intercept equal to 0). Deviation from the line of equality indicates a lack of agreement between the two methods. In the case at hand, the linear regression between the Hb concentration determined by UV–VIS and the concentration yielded by the reference method produced a straight line for which the least square regression equation is UV–VIS predicted = (0.0816 ± 0.1257) + (0.9688 ± 0.034) * (Hb), with a correlation coefficient 0.988. The resulting line is close to the line of equality: the 95% confidence intervals for the slope (0.935–1.0032) and the intercept (− 0.0441–0.2073) include respectively the values 1.0 and 0.0. Thus, the results provided by UV–VIS measurement are comparable to those produced by the CNMHb method. A paired test comparing the Hb concentrations obtained by the two methods was carried out to verify this result. For the 21 samples in the validation set, the mean of difference ${\overline{\text{X}}}$_d_ was 0.0115, and the standard deviation of the difference S_d_ was 0.455. According to these data, the value of t_cal_ = (${\overline{\text{X}}}$_d_ $\sqrt{\mathrm{n}}$)/S_d_ was 0.116. As t_cal_ is less than t_(95%, n−1)_, it can be confirmed that the UV–VIS method yielded results for Hb comparable with those achieved by the reference CNMHb method, with a significance level of 0.05. In fact, the mean recovery of the samples from validation by the UV–VIS determinations was 93.6% (see Table 1).

**Table 1:** Comparison of the analytical figures of merit (accuracy, precision, LOD, and linearity) for the different univariate and multivariate assayed methods.

Method	Univariate calibration		Multivariate calibration	
	UV–VIS	RGB 1 V	RGB PLSR	RGB MLF-ANN
*Precision	Variation coefficient (%)			
Hb 0.5 (g L^–1^)	1.18	4.67	25.0	5.56
Hb 2.5 (g L^–1^)	1.35	3.95	4.98	3.82
Hb 10 (g L^–1^)	1.05	1.10	2.56	0.10
**LOD (g L^–1^)	0.18	0.30	0.52	0.20
**LOQ (g L^–1^)	0.60	1.00	1.70	0.66
Linearity (g L^–1^)	0.18–10	0.30–10	0.52–10	0.20–10
Mean recovery (%)	93.6	86.1	102.1	102.3

*Precision was studied at three haemoglobin (Hb) concentration levels: 0.5, 2.5 and 10 g L^–1^. **LOD limit of detection, *LOQ* limit of quantification.

### RGB-univariate calibration method

In the case of RGB method, two different calibration approaches were applied: univariate calibration and multivariate calibration. When univariate calibration was tested for the four colour parameters extracted from images of samples in the calibration set, the values of G, B and intensity were not well correlated with the Hb concentration. The only colour variable exhibiting a linear relationship with the Hb concentration was the R variable (corresponding to red tones). This result was expected as the colour of the serum solutions are in the red range (from yellow-orange to intense red) (Fig. 1).

**Figure 4:**
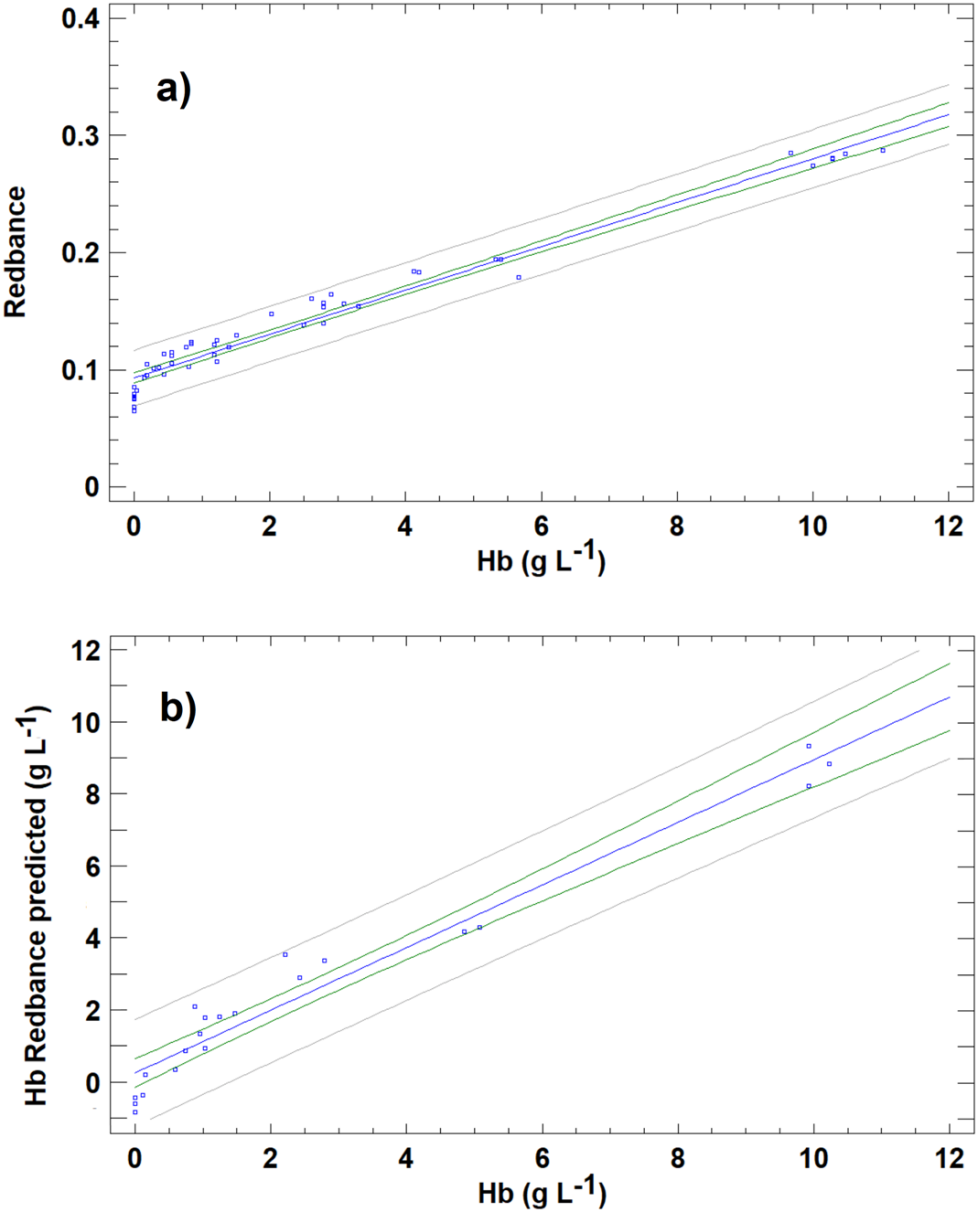
(**a**) Calibration curve for haemoglobin (Hb) concentration prepared from calibration set for the RGB univariate method (R-based). (**b**) Prediction of haemoglobin (Hb) concentration levels using the RGB univariate method (R-based) for the validation samples compared with those provided by the CNMHb method.

With the objective of presenting a calibration curve using an analytical signal similar to those obtained for UV–VIS method, the raw R analytical signal was transformed in Redbance (similar to absorbance), calculated as follows:

1 $$\mathrm{Redbance }=-\mathrm{ log}10 \frac{\text{R}}{{256}}$$

where R is the value of extracted R data for the sample considered, and 256 is the total number of RGB-channels in the red range. Using this magnitude for the analytical signal, Redbance varies between 0 for colorless solutions and 2.4 for the darkest red solutions. According to this analytical signal, the relationship between Redbance and the concentration of Hb in serum cattle samples followed a linear relationship, described by Redbance = (0.0929 ± 0.0021) + (0.0187 ± 0.0005) (Hb), with a correlation coefficient of 0.983. However, as deduced from these results and seen in Fig. 4a, the results were not as satisfactory as the UV–VIS results, exhibiting greater data dispersion for calibration samples. The regression line for the concentration of Hb determined and the reference value (Fig. 4b) is a straight line that differs slightly from the line of equality, and in fact, the confidence interval for the slope (0.824–0.914) does not include a value of 1.0. This leads to overestimation of Hb concentrations for values below 5% and to underestimation for values above 5%.

### RGB multivariate methods

In multivariate calibration, two different procedures were used to build the mathematical model for predicting the concentration of Hb in serum based on the four colour variables. The first was based on PLS regression, while the second used MLF-ANN.

#### RGB-PLSR method

**Figure 5:**
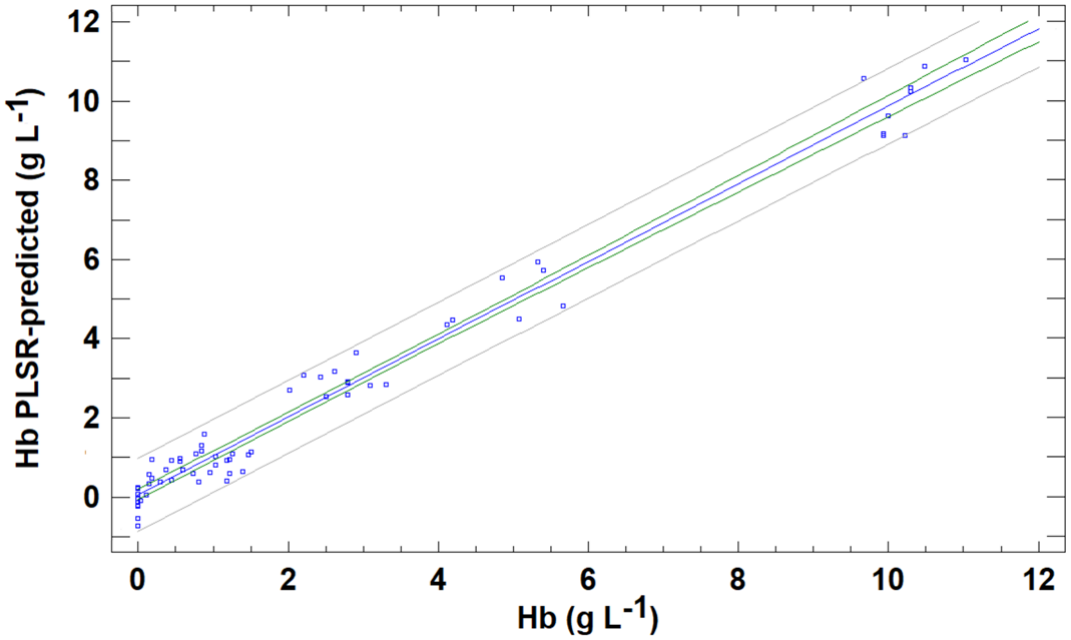
Predictions of haemoglobin (Hb) concentration levels using the RGB- PLSR method for the validation samples compared with those provided by the CNMHb method.

PLSR was applied to the 70 samples of data matrix with the four X-variables (R, G, B and weighted intensity) and the Y-response (Hb). Previous division between calibration and validation sets was deleted because in the present case, tenfold cross-validation was conducted as described in the methods section. The model was fitted to the training set composed of 90% of samples, and it was used to predict 10% of samples in the validation set. The process must be repeated 10 times in order to guarantee that all samples are included in the validation set at least once (this validation strategy evidently has a high computational cost; however, this is not problem for current computer processors and the modern chemometric packages that can automatize this procedure). In the present study, a three LF model rank was selected as optimum, retaining 99.9 and 98.1% of the total X- and Y-variance, respectively. The explained variance for the response in the prediction was 97.6%. The predicted values for the validation samples in relation to the reference Hb value are presented in Fig. 5. The results are clearly an improvement on the univariate approach. The linear equation obtained was: PLSR Predicted = (0.051 ± 0.0.069) + (0.9807 ± 0.0170) (Hb), R = 0.990. If the straight line obtained is compatible with the line of equality (with slope = 1 and intercept = 0), then this means that the RGB-PLSR method produces comparable results to the reference method. A test based on the definition of a joint confidence region for the intercept βo and the slope β1 (which is an ellipse) was applied to verify this endpoint. In the case at hand, the 95% confidence region obtained included values of βo = 0 and the β1 = 1. Then, with a confidence level of 95% RGB-PLSR method achieved compatible results with reference CNMHb method for haemoglobin measurement. Thus, the four colour variables together contain enough information to adequately predict the Hb concentration. In an attempt to enhance the predictive capacity of the RGB based-models, another multivariate calibration was constructed using a multilayer feed-forward artificial neural network.

#### RGB-MLF-ANN method

**Figure 6:**
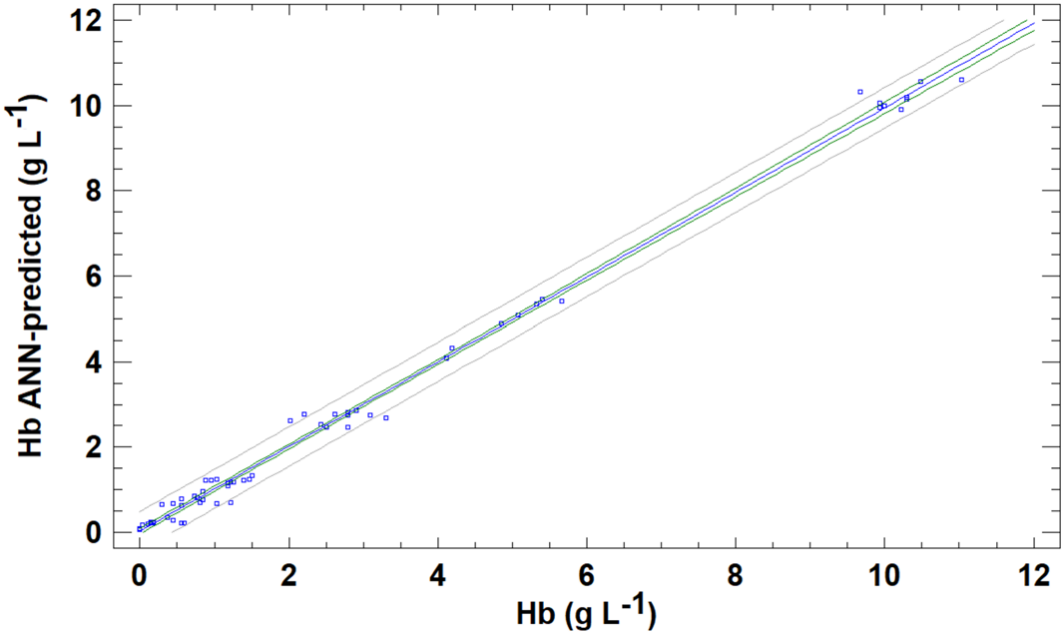
Predictions of haemoglobin (Hb) concentration levels using the RGB- MLF-ANN method for the validation samples compared with those provided by the CNMHb method.

In this section, an MLF-ANN was used to predict the Hb concentration on the basis of the raw data for the same four colour variables. The neural architecture for the MLF network was selected as follows. The input layer was composed of four neurons (a number equal to the number of input colour variables), and the output layer was formed by one neuron, yielding the Hb value predicted by the network. The number and size of the hidden layers were selected on the basis of minimum root mean square error (RSE) for the complete data set. The lowest RSE was achieved using a neural network with three layers (4–7-1) and was used for further calculations. Other network architectures with 2 hidden layers provided similar RSE, but the simplest structure with three layers was preferred. The transfer function used for output calculations was sigmoidal: f(x) = 1/(1 + [exp(–x)]). The initial weights of the connections between neurons were randomly selected in the range 3 to − 3. The adaptive learning rate parameter η and the momentum i (the parameters updating the weight of the connections after each epoch) were 0.2 and 0.5 respectively, and the maximum number epochs was limited to 500 to prevent overfitting. For validation, a tenfold cross-validation was applied as described above. The serum Hb concentrations predicted by MLF-ANN are presented in Fig. 6. The ANN provides even better results than PLSR. The comparison between ANN predicted values and reference Hb concentration was appropriate, following a fitted line ANN-predicted = (0.0270 ± 0.0348) + (0.9916 ± 0.0083) (Hb), with a correlation coefficient of 0.997. The compatibility with the line of equality was positively demonstrated using the same test as in the preceding section. Thus, the optimized neural network is capable of extracting useful information from raw colour data, correctly predicting the Hb concentration in serum samples, as determined by the reference CNMHb method.

### Analytical figures of merit

In addition to evaluating the accuracy expressed in terms of the predictive capacity of the methods described above, other analytical figures of merit, such as precision at three concentration levels, LOD and linearity, were tested. The results obtained are summarized in Table 1. The UV–VIS method produced adequate results relative to the CNMHb procedure. Thus, measurement of the absorbance at 540 nm is a rapid, simple method of estimating haemolysis in cattle serum. The LOD was the lowest of all procedures developed, and the linearity of the absorbance relative to the actual concentration of Hb was up to 10%. The RGB univariate method, based on the response in the red channels measured as Redbance, produced unsatisfactory results, because the predictions for validation samples overestimated the Hb content for low values and underestimated high values. This direct colour-based univariate method also produced poor results regarding precision (especially for concentration values lower than 1%). Thus, the RGB-multivariate approach improved the results obtained. The predictions obtained in both multivariate RGB methods based on PLSR and MLF-ANN calibrations produced similar results to the CNMHb method. However, the precision of the predictions by the PLSR method and consequently the recoveries by this method were very poor for low values. By contrast, calibration using a neural network yielded adequate results for precision, with CV in the range 0.10–5.56%. In addition, the linear response for RGB-ANN method yielded up to 10% of Hb, and the mean recovery compared with CNMHb method was 102.3%.

## Discussion

Two colour-based methods for determining haemolysis in bovine serum samples by measuring the concentration of Hb were tested. Both the direct UV–VIS spectrophotometric and RGB-MLF-ANN methods produced satisfactory results, providing results that were statistically comparable with those obtained by the CNMHb reference method (*P* < 0.05). In both cases, the figures of merit were adequate and haemolysis was detected at 0.50 g L^–1^, the minimum level that usually interferes in blood test results^17^.

Many methods of measuring Hb have been developed, as Hb is used to indicate anaemia and in vitro haemolysis, both of which are important and occur frequently in human medicine. Historically, Hb measurement has relied on well-equipped laboratories and use of chemical procedures such as the CNMHb method, the reference method for determining Hb concentrations according to the International Committee for Standardization in Haematology (ICSH)^27^. This approach is based in the measurement of the absorbance at 540 nm of the most stable derivate of Hb, cyanmethemoglobin^28^. However, the ICSH only recommends use of this method by national committees for the standardization of haematological methods or by official holders appointed by the government. Use of this method in the haemolysis test is not feasible as it is time consuming and produces toxic residues. Nevertheless, the method is still frequently used in some countries^29^.

Nowadays laboratories worldwide aim to systematically detect haemolysis in samples due to the high incidence and significance of this phenomenon in clinical pathology. As the procedure is intended to be performed in all types of laboratories, faster and simpler methods of detecting and quantifying haemolysis are being developed. Spectrometry is considered the best method of evaluating free Hb and, although the standard use of HI is proposed in multiple guidelines^2,30^, there is a generalized perception that this recommendation has not been fully assessed^19^. The HI calculates the degree of haemolysis by measuring absorbance of serum or plasma samples at different wavelength pairs (such as 570 and 600 nm or 660 and 700 nm), and this method is now incorporated in most large workstations. A number of spectrometric methods use two or more wavelengths in jaundice or lipemic samples to prevent the interference observed in single wavelength results.

In a classic study, Malinauskas et al.^31^ used bovine samples to compare nine different spectrophotometric methods involving the use of two and three combined wavelengths and two other chemical methods to measure Hb in plasma. These authors concluded that spectrometric methods were safer, easier and more precise and accurate than methods involving addition of chemicals. These different approaches are possible because Hb absorption spectra enable estimation of Hb at a broad wavelength range, with at least two main characteristic maximums at around 420 and 540 nm^32^. The 540 nm peak was chosen for designing the UV–VIS direct spectrophotometric method in this study because good results have been obtained in other studies for assessing haemolysis in humans and other animal species^7,33–35^. Other wavelengths close to these main peaks have also been used. In human medicine, a spectrophotometric approach based on λ = 414 nm for measuring low levels of haemolysis in serum was demonstrated to be a reliable method of identifying haemolytic samples^36^. In this case, the proposed method enabled detection of 0.004% haemolysis. Other authors who have studied the potential variations in absorbance peaks of Hb on the basis of the partial pressure of oxygen (PO_2_) concluded that when the PO_2_ is higher than 100 mm Hg, then the peak at 576 may also be useful^37^. Only slight differences in the absorption spectra of human and bovine oxyhaemoglobin are found^38^, and therefore the analytical technique and the wavelength used in these studies should also be valid in bovine patients, as demonstrated for the UV–VIS method presented here. In this first method, univariate calibration of the absorbance at 540 nm vs. In our experience Hb content was sufficient for good prediction of haemolysis in the bovine serum samples. However, some weaknesses of the one-wavelength approach developed include the following aspects: (1) the calibration carried out with a single wavelength might not be able to distinguish whether the analytical signal is only from the target analyte (Hb in this case) or from some interferent present in the matrix (serum) that can increase or decrease the measured signal; and (2) the difficulty in carrying out field measurements and the potential interference from the change in serum colour in lipemic and icteric samples. In spite these considerations, on the basis of the results obtained, the procedure applied here based on one single wavelength is considered appropriate because, in addition to having been validated, it is a simple, rapid economic, non-destructive and available technique for measuring haemolysis in cattle serum samples.

Bearing in mind that spectrophotometric methods may not be feasible for professionals in field conditions or for small veterinary laboratories, a second method based on RGB data extracted from a digital image of samples obtained with a simple digital device was developed. Implementation of this method on a mobile phone or other mobile device would provide a valuable tool for field professionals in order to verify the quality of the sample before sending it to specialized laboratories. Of the different approaches evaluated, the multivariate RGB-MLF-ANN method produced the best results for the determination of Hb. Although the analytical figures of merit for RGB-MLF-ANN were slightly poorer than those yielded by the UV–VIS method (lower precision for 2.5% haemolysis and slightly higher LOD), the results obtained by this procedure were also acceptable and similar to those obtained by the CNMHb method (*P* < 0.05). The method can therefore be used for reliable measurement of haemolysis. Common tools such as digital cameras and phones are increasing being used in the scientific field as these relatively inexpensive instruments include the hardware and software required for such tasks. RGB data have been used to accomplish diverse analytical tasks such as detecting adulterations in old wine^23^ and determining iron contents in blood, wine and water^39^. In veterinary science, cameras that provide RGB data enable the development of methods of identifying individual cows^40,41^, measuring and controlling the heartbeats of cattle^42^, dividing flesh and viscera in poultry^43^ and detecting chicken breast spoilage^44^, among others. In the medical field, a variety of devices use spectrometry or RGB data for determining Hb in humans, in both invasive^45,46^ and non-invasive methods^47–50^. Most of these instruments and devices were designed for detecting anaemia in human patients, but RGB phone-based methods have also been applied to detect in vitro and in vivo haemolysis^51^. Archibong et al.^52^ used RGB values to detect in vivo haemolysis with a mobile phone method, with an accuracy of 0.01 g L^–1^ of Hb in plasma samples and a correlation coefficient of R^2^ = 0.9703. Lopes et al.^53^ developed a method involving computer vision and neural networks (NN). After training the NN, values of 0.4 g L^–1^ of Hb were determined. The device estimated haemolysis with sufficient accuracy to guide laboratory decision in the blood test pre-analytical stage. The use of NN provides advantages for data interpretation regarding nonlinearity, supervised learning, adaptability and context information. For these reasons, NNs have been used as a multivariate prediction tool for evaluating sample haemolysis as well as other well known calibration approaches, such as partial least squares regression (PLSR). Thus, Kim et al.^54^ satisfactorily used PLRS to quantify blood Hb levels by a reconstructed reflectance spectra from RGB data obtained from cattle third eye with a three-colour sensor camera. In this study, the authors also determined the correlations for the R channel only, for determining blood Hb, concluding that although this channel was associated with Hb (*P* = 0.047), the correlation coefficient was too low. This finding supports that idea that although R-channel may be related to Hb content, application of a multivariate approach is required to produce consistent, robust results. In the RGB univariate method evaluated here, the use of the R-channel only (because the relationship with Hb is expected due to the red colour) was also explored. However, although the results were better than those obtained by Kim et al.^54^ (R^2^ = 0.973 for direct R-channel procedure compared with R^2^ = 0.260 for the R-channel procedure), the lack of adequate precision (with a high data dispersion for calibration samples) as well as the overestimation of Hb concentrations for values lower than 5% and underestimation for values higher than 5% rule out the use of this method. The approach involving the use of a multilayer neural network with forward learning could be used to develop new procedures based on low-cost cameras (with higher resolution than the used in this work), or even mobile phone applications to detect and quantify hemolysis in cattle from digital images. This will provide veterinary professionals working in the field with a simple, fast and easy-to-use tool for rapid estimation of the degree of hemolysis in bovine samples.

## Data Availability

The datasets generated during and/or analyzed during the current study are available from the corresponding author on reasonable request.
